# The absence of one’s intimate partner promotes dyadic competition through enhanced interbrain synchronization between opponents

**DOI:** 10.3389/fpsyg.2024.1298175

**Published:** 2024-01-24

**Authors:** Shuyu Jia, Yujia Meng, Yuan Gao, Lihong Ao, Lei Yang, He Wang, Yingjie Liu

**Affiliations:** ^1^School of Psychology and Mental Health, North China University of Science and Technology, Tangshan, Hebei, China; ^2^School of Public Health, School of Psychology and Mental Health, North China University of Science and Technology, Tangshan, Hebei, China

**Keywords:** EEG, hyperscanning, competition, intimacy, gender

## Abstract

Competition is a common occurrence in life, but the influence of intimate relationships on people’s competitiveness remains unknown. Grounded in Darwin’s theory of sexual selection, this study utilized EEG hyperscanning technology to investigate the influence of the presence of romantic partners and the gender of competitors on the interbrain synchronization of female individuals during competitive contexts. The research results showed that in competitive interactions, there was a significant increase in Theta and Alpha frequency band activity between females and their competitors. Interbrain synchronization was strongest when their partners were not nearby and females competed with same gender competitors. The research results indicate that intimate companionship has an impact on the early alertness and late cognitive execution mechanisms of female individuals in competition, and due to intimate relationships, females pay more attention to same-gender competitors. This study demonstrates that the presence of intimate partners can affect a female’s competitive state and brain synchronization with opponents of different genders, improving the theoretical explanation of intimate relationships and competitive interactions.

## 1 Introduction

With the rapid development of modern society, competition has become a hot topic in the field of social neuroscience. Competition stimulates the creative spirit and affects people’s lives and the development of intimate relationships (Chuang et al., 2015). Darwin described two types of sexual selection: the law of battle, according to which competition occurs between participants of the same gender (usually male) for access to mates, and the taste of beauty, which refers to (typically female) preference for partners based on specific innate traits (Darwin, 1981). Humans also exhibit mate choice competition in intimate relationships. Females often choose the ideal spouse based on the results of male–male competition, with such competition mainly occurring between participants of the same gender. A thorough exploration of the regulatory role of intimate relationships in competition plays a crucial role in fostering positive interpersonal dynamics, facilitating a deeper understanding of intimate relationships, and promoting their development. It can provide valuable guidance for fostering healthy interpersonal relationships and psychological well-being.

Recent studies have also demonstrated competition between the genders. With the development of society, competition between opposite gender participants increased, and the competitive scenarios that females face have altered accordingly (Semenyna et al., 2020). A study on archers showed that females exhibit higher levels of arousal and cognitive anxiety than males at different stages of competition (Abdoshahi et al., 2023). When participants compete, females are more sensitive to competition. In addition, females generally have higher awareness of and influence over interpersonal communication processes (Acitelli, 1992). They tend to be more enthusiastic and submissive than males, while males tend to be more dominant and indifferent than females (Suh et al., 2004). There are significant differences between males and females in interpersonal communication. Competition is an interactive process, and regardless of relationships, humans inevitably engage in social interaction with others; intimate relationships are no exception (Li et al., 2023). Previous studies have shown that people are more sensitive to interpersonal factors in more intimate (such as romantic) relationships, and females generally exhibit higher interpersonal sensitivity (Lambert and Hopwood, 2016). Therefore, female participants may be influenced by intimate relationships to a greater extent in competitive interaction scenarios.

Neural synchrony is a characteristic of interpersonal relationships, but the cognitive neural mechanisms underlying how intimate relationships influence females’s competition remain unclear. Previous theories have suggested that an participant’s efforts in competitive interactions can result in a certain degree of overlap between their brain activity and that of their interaction partner (Gugnowska et al., 2022; Chang et al., 2023). There is evidence that when participants receive positive feedback in competitive contexts, compared with cooperative contexts, the Theta band activity in the frontal lobe exhibits greater interbrain synchronization. With the improvement of subjective consciousness, the interbrain synchronization of participants in a competition decreases (Balconi and Vanutelli, 2018). The relationship between interbrain synchronization and external performance demonstrates the presence of a link between cognitive ability and the Theta frequency band, which plays a role in interbrain synchronization. In addition, Putri et al. (2022) found that in competitive interactions, the winner may monitor the opponent’s activity. Alpha frequency band activity is related to participant perception and consciousness. Additionally, research has revealed that the interbrain synchrony of individuals is influenced by the synchronization of competitive behavior and also by the dynamics within the competitive group (Gamliel et al., 2021). In other words, when individuals compete within their own group as opposed to competing with an external group, their behavior and brain synchronization are higher. This suggests a close association between interbrain synchrony and the interaction within the competitive group.

In a study examining the impact of fairness and competition on brain activity, the findings revealed that compared to males, females are more influenced by short-term induced affective preferences, exhibiting greater differences in brain activity components such as FRN and P3 in response to generally liked fair competition and disliked unfair competition (Wang et al., 2014). This suggests that the brain activity of females during competition may be more regulated by social contextual factors such as interpersonal relationships. Moreover, previous studies have shown that people are more sensitive to interpersonal factors in more intimate (such as romantic) relationships, and females generally exhibit higher interpersonal sensitivity (Lambert and Hopwood, 2016). Thus, we speculated that female participants may also be influenced by intimate relationships to a greater extent in competitive interactions. Interbrain synchronization between participants can reveal the neural mechanisms underlying competitive interactions in females and explore the impact of intimate relationships on competition. In summary, we hypothesized that intimate relationships would influence interbrain synchronization between female participants and their competitors in competitive contexts. Specifically, females in the presence of their intimate partners may experience reduced interbrain synchronization with their competitors due to heightened sensitivity to interpersonal relationship dynamics.

Electroencephalography (EEG) is used to detect dynamic oscillation activity with high temporal resolution (i.e., at the millisecond level) and has become one of the most commonly used techniques in hyperscanning research (Balconi and Vanutelli, 2018). Brain synchronization, as indicated by coordinated and synchronized brain activity between two or more individuals, is a hallmark of real-time social cognition, including joint attention and shared intention understanding (Zhou et al., 2023). Brain synchronization may occur during social interactions, communication, or joint engagement in activities, and it plays a role in empathy, understanding others’ perspectives, and establishing social relationships. Furthermore, it is likely influenced by emotional connection and social dynamics (Djalovski et al., 2021). Phase Locking Value (PLV) is one of the indicators used to describe the degree of phase synchronization between different frequencies in EEG signals. Compared with commonly used brain functional connectivity indicators such as Phase lag index (PLI) and Weighted Phase-Lag Index (wPLI), it can more accurately describe the degree of phase synchronization between different frequencies without being affected by signal amplitude (Ma et al., 2022). Compared to studying individual brains in isolation, the use of hyperscanning research provides a more comprehensive and integrated approach that helps us gain a deeper understanding of the impact of intimate relationships on the brain synchronization state of female participants during competitive interactions and competition with opponents of different genders (Czeszumski et al., 2020). This study used EEG technology to explore the cognitive and neural effects of the presence of one’s partner and opponent gender on interbrain synchronization during female competition through hyperscanning. This study supplements the field of female competitive psychology and brain activity research and expands upon theoretical explanations of intimate relationships and competition.

## 2 Method

### 2.1 Participants

We used GPower 3.1 (Faul et al., 2007) to determine the sample size needed. The parameters were as follows: *f* = 0.4, *α* = 0.05, 1–β = 0.8, repeated-measurements analysis of variance, and intertest factor. For these parameters, the minimum sample size was estimated to be *N* = 52, for a total of 104 people, that is, 13 pairs of subjects in each condition. A group of 120 healthy university students (30 males, 90 females, age range: 18–25 years, mean age: 20.30 years) participated in the study. In the experiment, participants were randomly divided into 60 dyads. A total of 60 pairs (dyads) of participants were formed, including 30 participants with their intimate partners nearby (15 dyads of same gender competition, 15 dyads of opposite gender competition) and 30 participants with their intimate partners absent (15 dyads of same gender competition, 15 dyads of opposite gender competition). Members of pairs were not acquainted with each other before the experiment. All participants were right-handed, with normal or corrected-to-normal vision and hearing as well as no self-reported history of neurological/psychiatric disorders or drug abuse; participants were unaware of the experiment’s purpose. The participants in a relationship reported being intensely in love (duration of “being in love”: *M* = 15.15 months, range = 2–36 months). After the experiment, participants were paid 10 to 20 yuan. The medical ethics committee of the local schools approved the study, which was conducted in accordance with all provisions of the Declaration of Helsinki. All participants signed informed consent forms.

### 2.2 Tasks and procedures

The experiment consisted of a visual cue-target task (Cui et al., 2012; Cheng et al., 2015). Let the participants sit 50 cm in front of the computer display. Among the lovers with uniform seats, the female is on the left, the competitor is on the right, and the female accompanies the lover on the left. In the condition that the lover is not accompanied, the position of the lover’s companion is replaced by the stranger to eliminate the influence caused by mere gaze.

First, the experimental requirements were explained to the participant, and corresponding guidance was presented on the screen. Before the formal experiment began, there were five practice sessions to familiarize participants with the competitive task process. At the beginning of each trial in the formal experiment, a 2,000 ms black screen was first displayed, followed by a grey circle with a duration of 600 ms to 1,500 ms in the center of the screen, followed by a green circle for target stimulation. Both participants had to press their respective buttons on the keyboard when seeing the target stimulus. The target stimulus remained on the screen until both participants responded. Afterward, 4 s of feedback appeared.

**Figure 1:**
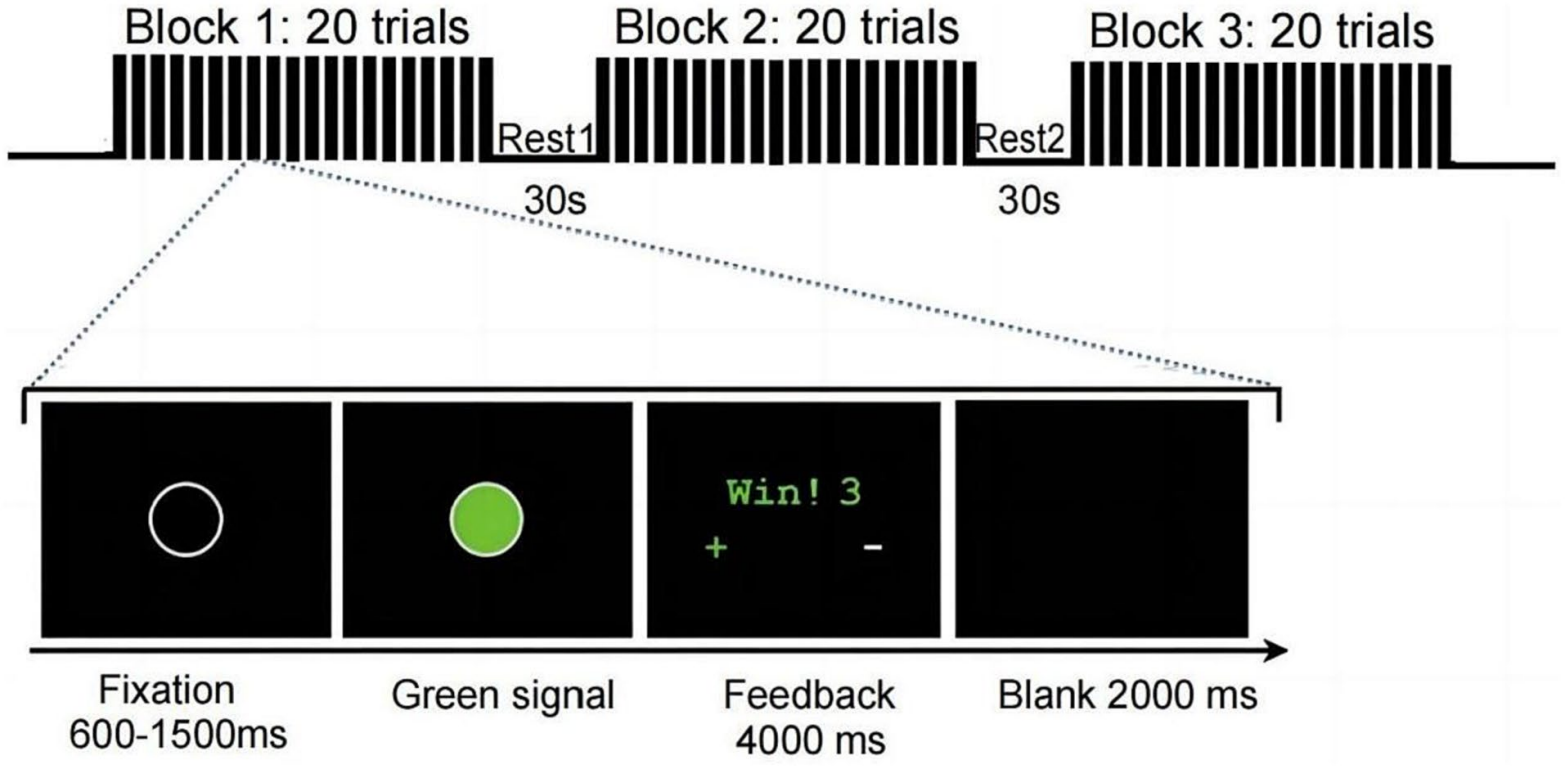
Experimental design.

In the task, the original base score for both sides was 100 points, with the winner adding 3 points in each round and the loser subtracting 3 points from their totals. After both parties pressed the button, the feedback screen displayed the winning or losing situation in the current competitive task and the participant’s respective points and accumulated points. After a black screen was presented for 2,000 ms, the next attempt was initiated. The formal experiment consisted of 3 blocks, each containing 20 trials, for 60 blocks, as shown in Figure 1.

### 2.3 Subjective measurements

After the experiment, the female participants in the couples were asked to complete the Chinese version of the Interpersonal Reactivity Index-C (IRI), which included four subscales: perspective selection, personal pain, imagination, and empathy. A total of 22 questions were scored with Richter’s five points (0–4, from “inappropriate” to “very appropriate”) (Pang et al., 2022). In addition, we collected each participant’s attitudes toward her partner and the experiment; this information included: (1) the Competitor’s ability; (2) The good-looking degree of the opponent. Participants used a 9-point Likert scale, which ranged from 1 (“not very much”) to 9 (“very much”) for all ratings. No discussion was allowed while rating.

### 2.4 Data acquisition

Referring to the EEG hyperscanning equipment used by Barraza et al. (2019), two 64-channel SynAmps2 EEG recording systems produced by the Neuroscan Company were used to record the EEG signals of two participants simultaneously. Ground electrode, GND, referred to REF online. Using the international 10–20 system to record EEG signals, both vertical and horizontal electromyography (VEOG and HEOG, respectively) are bipolar recordings, with a filter bandpass of 0.05 Hz to 100 Hz and a continuous sampling frequency of 1,000 Hz. The impedance among all electrodes and the scalp was less than 5 KΩ. At the same time, participant response times (from the initial presentation of stimuli to the participant’s button response) were recorded through E-prime 3.0.

### 2.5 Data analysis

#### 2.5.1 Behavior performance

Response times (RTs) and winning trials were recorded for each participant dyad. In order to measure the closeness of participants to complete tasks in the experiment, and to avoid Outlier in the data, calculate the median of the absolute value of the response time difference of each pair of participants in all trials, that is, the median difference in response time, the median DRT. The smaller the value, the closer the response time of participants is:

$\text{DRT}=\text{abs}\;\left(\text{RT}1-\text{RT}2\right)$

To quantify the subjects’ performance, we calculated the overall percentage of winning trials (PWT) (Cui et al., 2012; Cheng et al., 2015). First, the variables were checked for normality via Kolmogorov–Smirnov tests, and the median-DRT and PWT are by a normal distribution. This study uses univariate analysis of variance to analyze median-DRT with partner status and opponent gender as fixed variables, α The level is set to 0.05. At the same time, the PWT was also investigated by univariate ANOVA with partner status and opponent gender as fixed variables. Finally, Spearman bivariate correlation analysis was performed to test the relationship between brain synchronization and behavior.

#### 2.5.2 EEG data

The data analysis software was MATLAB R2018b. The data preprocessing process included locating electrode points, deleting useless electrodes (HEGO, VEOG), using the whole brain average as a reference point, performing 1 Hz high pass filtering and 45 Hz low pass filtering, down sampling to 500 Hz, taking the time point of stimulus occurrence as zero, analyzing segments with a time range of −2,000 ~ 1,000, performing independent component analysis, and removing wave amplitudes exceeding ±100 uV artifact test number. The PLV was used to indicate neural synchronization, and PLV calculations were performed on everyone’s EEG data (Lachaux et al., 1999). Simply put, this method calculates the brain phase difference between all electrode pairs within a time window and then evaluates the stability of this phase difference through all experiments (Burgess, 2013). Current source density (CSD) analysis was performed on the pre-processed data before calculating the PLV to avoid volume conduction effects. Subsequently, the time-frequency matrix (window length, 400 ms; step, 1 Hz) of all segmented stored phase information was obtained using a short-time Fourier transform. Then, we determined the difference in the phase matrix of the two electrodes of each section and set φi and φj as the positive vector of the signal phase of the two electrodes, i and j, between the time window t and the frequency f. The phase difference between the two electrodes is:

$\varphi \;\text{ij}=\varphi i\cdot \varphi j\times \left(\varphi j\times \text{being}\;\text{the}\;\text{complex}\;\text{conjugate}\;\text{of}\;\varphi j\right)$

For each time-frequency point, we calculated the consistency of the phase differences of all segments at that time-frequency point and obtained the PLV of each time-frequency point as follows:

$\text{PLVij}=\text{abs}\left(1/N\times \sum \phi ij\right)$

Where N is the number of tests, we calculated the PLV of each pair of electrodes (i, j) between two participants (Electrode I was used for female participants, while electrode j was used for competitors). The PLV ranged from 0 to 1, where 0 indicated that the two signals were unsynchronized, and 1 indicated perfect synchronization.

**Figure 2:**
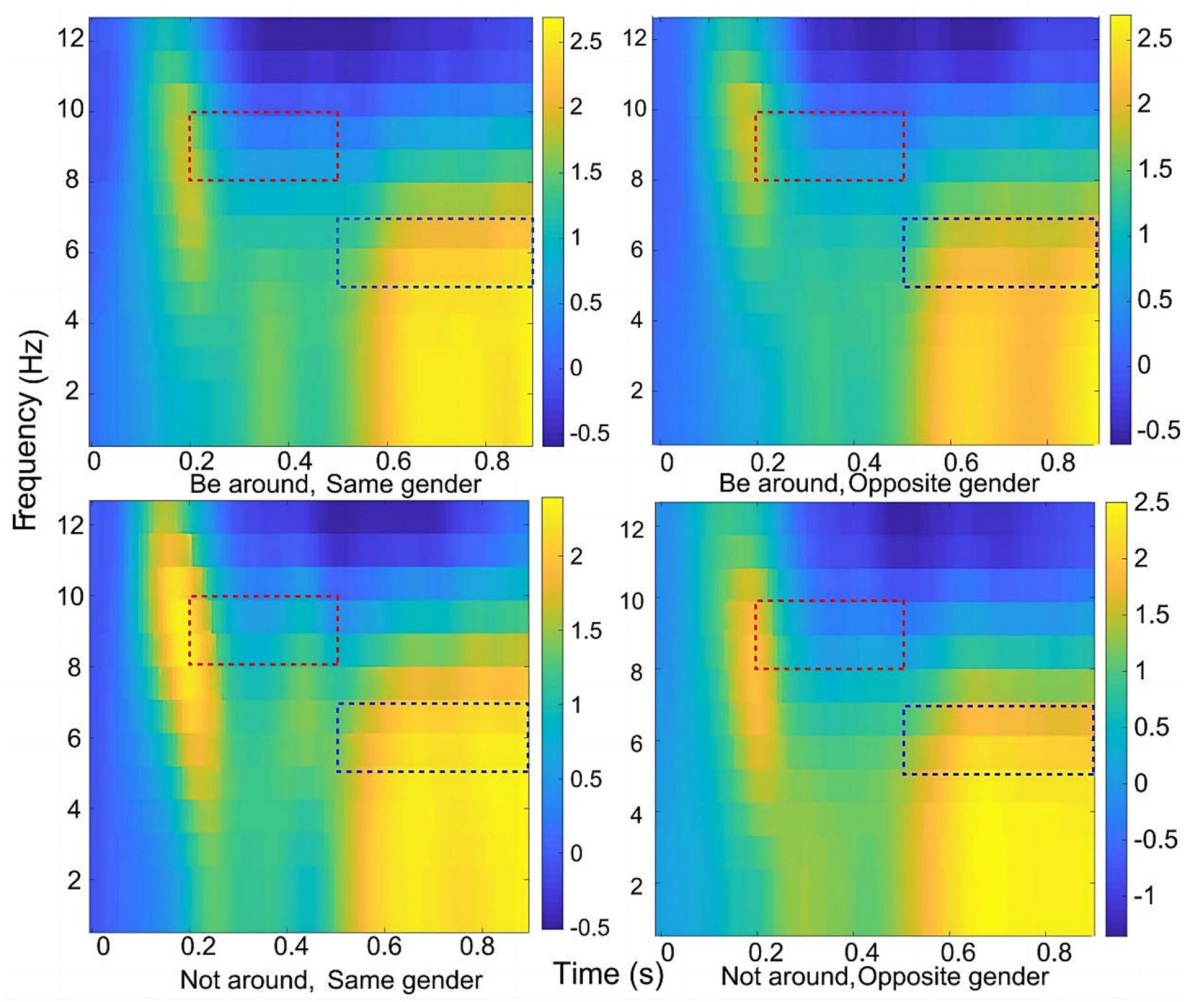
Mean time-frequency chart of the whole brain. The red box indicates the time period during which there is a significant difference in brain activity in the Alpha frequency band, while the blue box indicates the time period during which there is a significant difference in brain activity in the Theta frequency band.

The range of −800 to −200 ms before stimulation was chosen as the baseline correction window. Based on previous literature and the time-frequency maps generated by the average of the entire brain electrode points in this study (Figure 2), the range of −200–1000 was selected as the time period of interest, and the Theta frequency band (3–7 Hz) and Alpha frequency band (8–12 Hz) were defined as the frequency band of interest (Riddle et al., 2020; Abbasi et al., 2023). Using self-developed MATLAB code, a data-driven approach was employed to analyze the PLV between all electrode pairs in each 200 ms time segment. Independent sample t-tests, false discovery rate (FDR) correction, analysis of variance (ANOVA), and correlation analysis were conducted to examine the statistical significance and relationships.

## 3 Results

### 3.1 Behavioral performance

Two-way ANOVAs were performed for median DRT. We found that when the partner was not nearby, the IRI total score scale (*M* = 72.57; *SD* = 8.89) was significantly higher than that when the partner was nearby (*M* = 64.00; *SD* = 11.36) [*F*(1, 56) = 10.53, *p* = 0.002, $\eta_{p}^{2}$ = 0.158]. There were no significant effects of opponent gender or the partner presence × opponent gender interaction.

**Figure 3:**
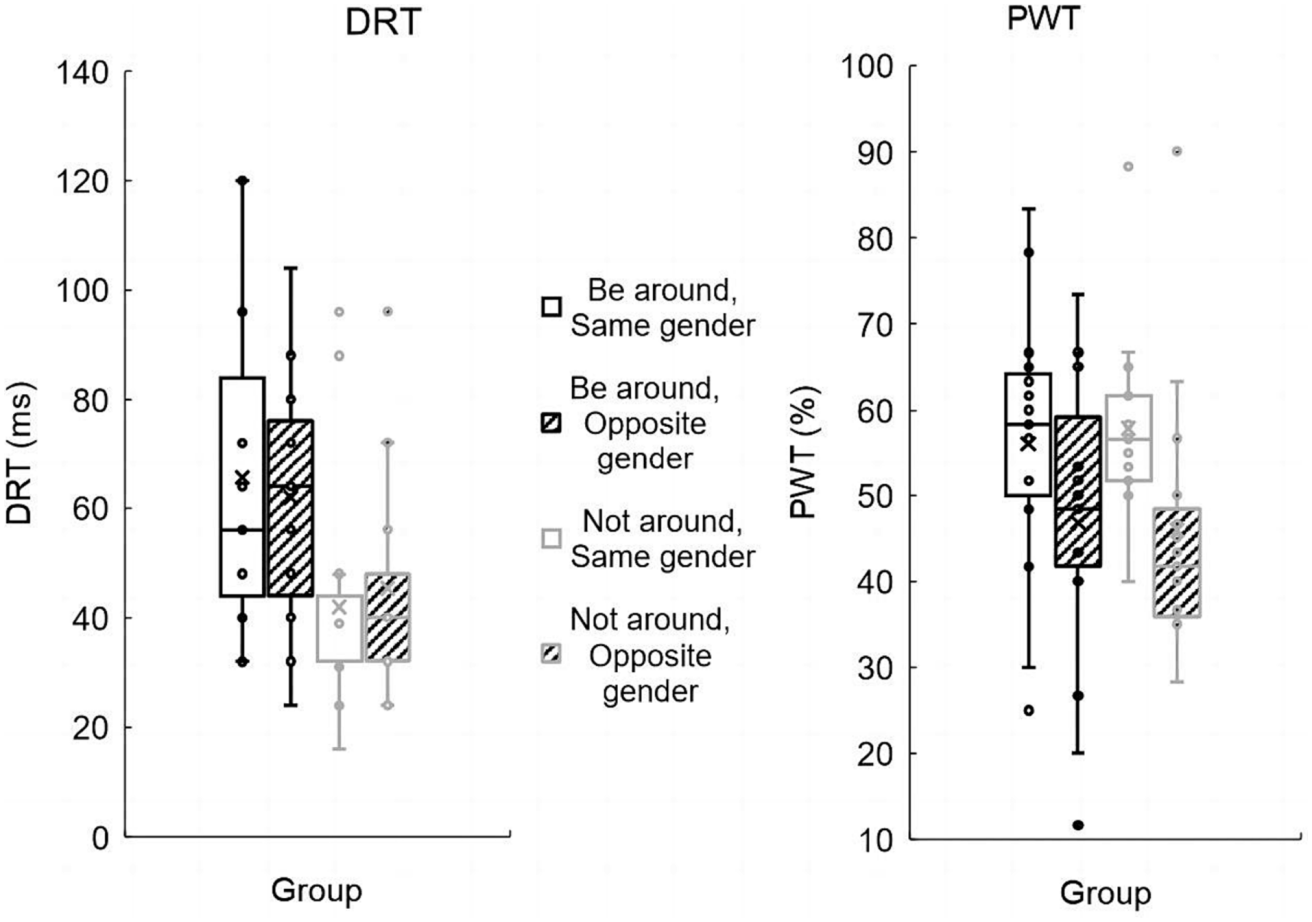
A boxplot representation of behavior results. The left side represents the behavioral results of DRT, and the right side represents the behavioral results of PWT. The 25th and 75th quartiles and the “whisker” showing the 5th and 95th percentile.

A univariate analysis of variance on the PWT was conducted. The results showed a significant main effect of dyad gender [*F*(1, 56) = 7.59, *p* = 0.008, $\eta_{p}^{2}$ = 0.12], with the PWT of same gender dyads (*M* = 57.00; *SD* = 2.74) significantly higher than that of opposite gender dyads (*M* = 46.33; *SD* = 2.74). The main effect of partner presence was not significant [*F*(1, 56) = 0.01, *p* = 0.909], and the interaction between partner presence and dyad gender was not significant [*F*(1, 56) = 0.12, *p* = 0.732] (see Figure 3).

### 3.2 Interbrain synchronization

The results of interbrain synchronization showed that during competition, interbrain synchronization between participants was significant in the Alpha and Theta frequency bands (Figure 2).

#### 3.2.1 Interbrain synchronization in the Theta band

First, *t* tests and FDR correction were performed for different groups. The results showed that when competing with an opponent of the opposite gender, there was a significant difference in interbrain synchronization between the FC5 and PO4 electrodes at 5–7 Hz (the Theta frequency band) in the period of 500–700 ms regardless of partner presence [*t* (17.043) = −3.02, *p* = 0.008, Cohen’s d = −1.10] (Cohen, 1988). When competing with an opponent of the opposite gender, interbrain synchronization (*M* = 0.11; *SD* = 0.04) was more significant when the partner was absent (*M* = 0.08; *SD* = 0.01). During 700–1,000 ms, when the partner was absent, regardless of opponent gender, there was a significant difference in interbrain synchronization at 5–7 Hz (the Theta frequency band) at the F8 and FC5 electrodes [*t* (28) = 4.32, *p* < 0.001, Cohen’s d = 1.58]. That is, when the partner was absent, the interbrain synchronization of same gender dyads (*M* = 0.10; *SD* = 0.02) was significantly greater than that of opposite gender dyads (*M* = 0.07; *SD* = 0.01).

**Figure 4:**
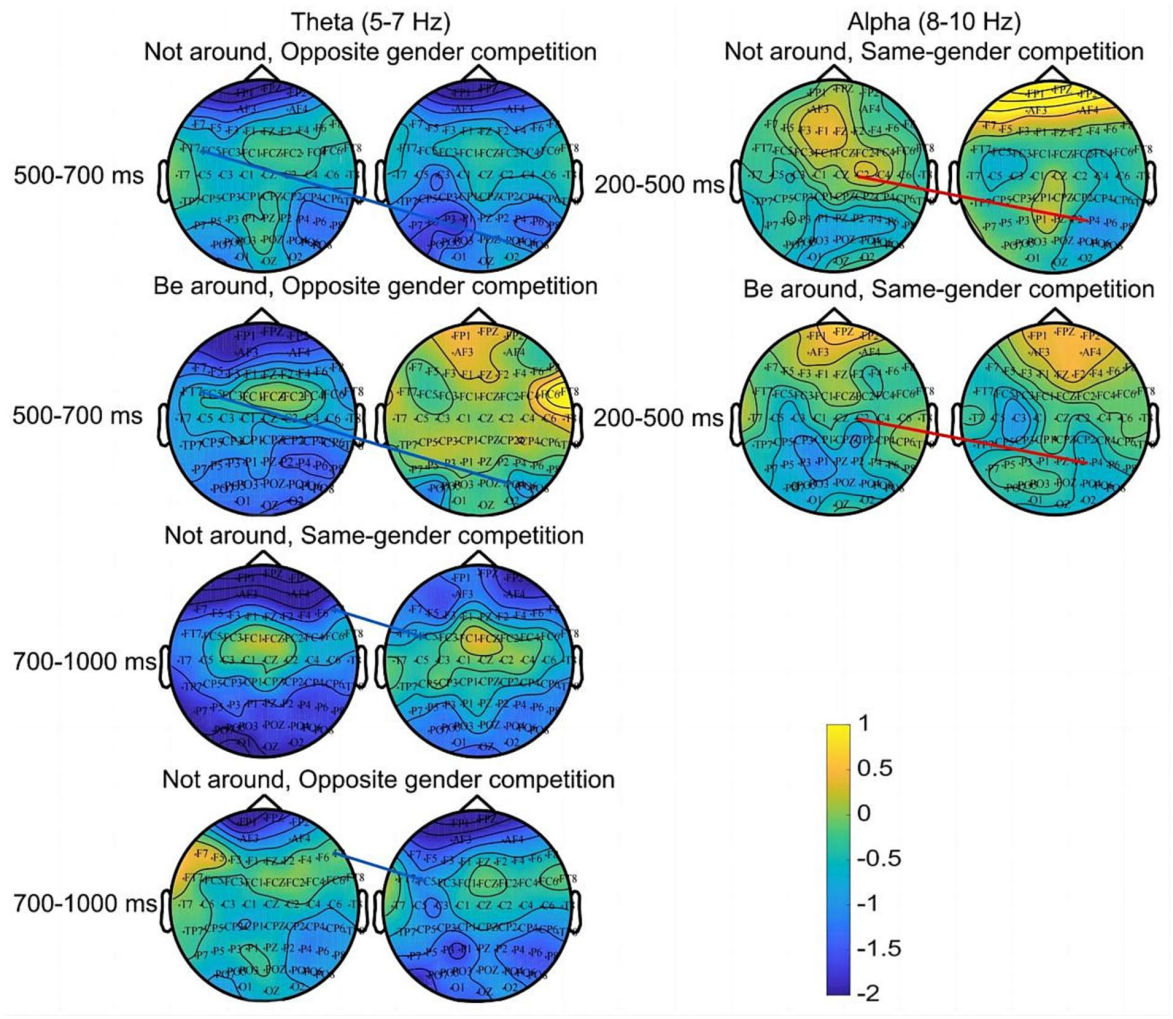
Interbrain synchronization. Lines connect pairs of electrodes displaying significant interbrain synchronization between members of dyads (*p* < 0.05). The color indicates the intensity of time-frequency activation. The red line represents the connectivity of brain synchronous electrode pairs in the Alpha frequency band, while the blue line represents the connectivity of brain synchronous electrode pairs in the Theta frequency band.

The analysis of variance showed that the main effect of partner presence was significant between the FC5 and PO4 electrodes [*F*(1, 28) = 9.53, *p* = 0.003, $\eta_{p}^{2}$ = 0.15]. When competing with an opponent of the opposite gender, the interbrain synchronization (*M* = 0.11; *SD* = 0.04) was higher when the partner was absent (*M* = 0.08; *SD* = 0.01). The main effect of opponent gender was significant between the F8 and FC5 electrodes [*F*(1, 28) = 10.19, *p* = 0.002, $\eta_{p}^{2}$ = 0.15]. That is, when the partner was absent, the interbrain synchronization of same gender dyads (*M* = 0.10; *SD* = 0.02) was significantly greater than that of opposite gender dyads (*M* = 0.07; *SD* = 0.01) (Figure 4).

#### 3.2.2 Interbrain synchronization in the Alpha band

The results of *t* tests and FDR correction showed that when competing with an opponent of the same gender, activity in the Alpha band (at 8–10 Hz) significantly differed between the C2 and P4 electrodes according to partner presence in the period of 200–500 ms [*t* (28) = −4.16, *p* < 0.001, Cohen’s d = −1.52]. When competing with an opponent of the same gender, the interbrain synchronization when the partner was absent (*M* = 0.13; *SD* = 0.03) was significantly higher than that when the partner was present (*M* = 0.09; *SD* = 0.02).

Univariate analysis of variance showed that the main effect of partner presence was significant between the C2 and P4 electrodes [*F*(1, 28) = 13.91, *p* < 0.001, $\eta_{p}^{2}$ = 0.20]. When competing with an opponent of the same gender, the interbrain synchronization when the partner was absent (*M* = 0.13; *SD* = 0.03) was significantly greater than that when the partner was nearby (*M* = 0.09; *SD* = 0.02). Moreover, the interaction between opponent gender and partner presence was significant between the C2 and P4 electrodes [*F*(1, 28) = 5.57, *p* = 0.022, $\eta_{p}^{2}$ = 0.09]. Further analysis showed that when the partner was absent, the interbrain synchronization of same gender dyads was significantly greater than that of opposite gender dyads (*M*_same gender_ = 0.13, *SD* = 0.01; *M*_opposite gender_ = 0.11, *SD* = 0.01; *p* = 0.012). When competing with an opponent of the same gender, the interbrain synchronization when the partner was absent was significantly greater than that when the partner was nearby (*M*_absent_ = 0.13, *SD* = 0.01; *M*_present_ = 0.10, *SD* = 0.01; *p* < 0.001) (Figure 4).

### 3.3 The relationship between the PLV and behavior

To evaluate the association between interbrain synchronization and motor response, a bivariate Spearman correlation analysis of the relationship between the PLV values and the median DRT of the dyads was performed. The correlation results showed that in the Alpha frequency band during 200–500 ms, in the same gender dyads, the interbrain synchronization of the C2-P4 electrodes was significantly negatively correlated with the median DRT (*r* = −0.46, *p* = 0.011). In the Theta frequency band (5–7 Hz) during 700–1,000 ms, when the partner was absent, the interbrain synchronization of the F8-FC5 electrodes was significantly negatively correlated with the median DRT (*r* = −0.41, *p* = 0.026). In the Theta frequency band (5–7 Hz) during 500–700 ms, in the opposite gender dyads, the interbrain synchronization of the FC5-PO4 electrodes was significantly negatively correlated with the median DRT (*r* = −0.46, *p* = 0.010). The above results indicate that the shorter the median DRT was, the closer the task completion time, the more intense the competition, and the higher the interbrain synchronization between subjects.

### 3.4 Subjective measurements

**Figure 5:**
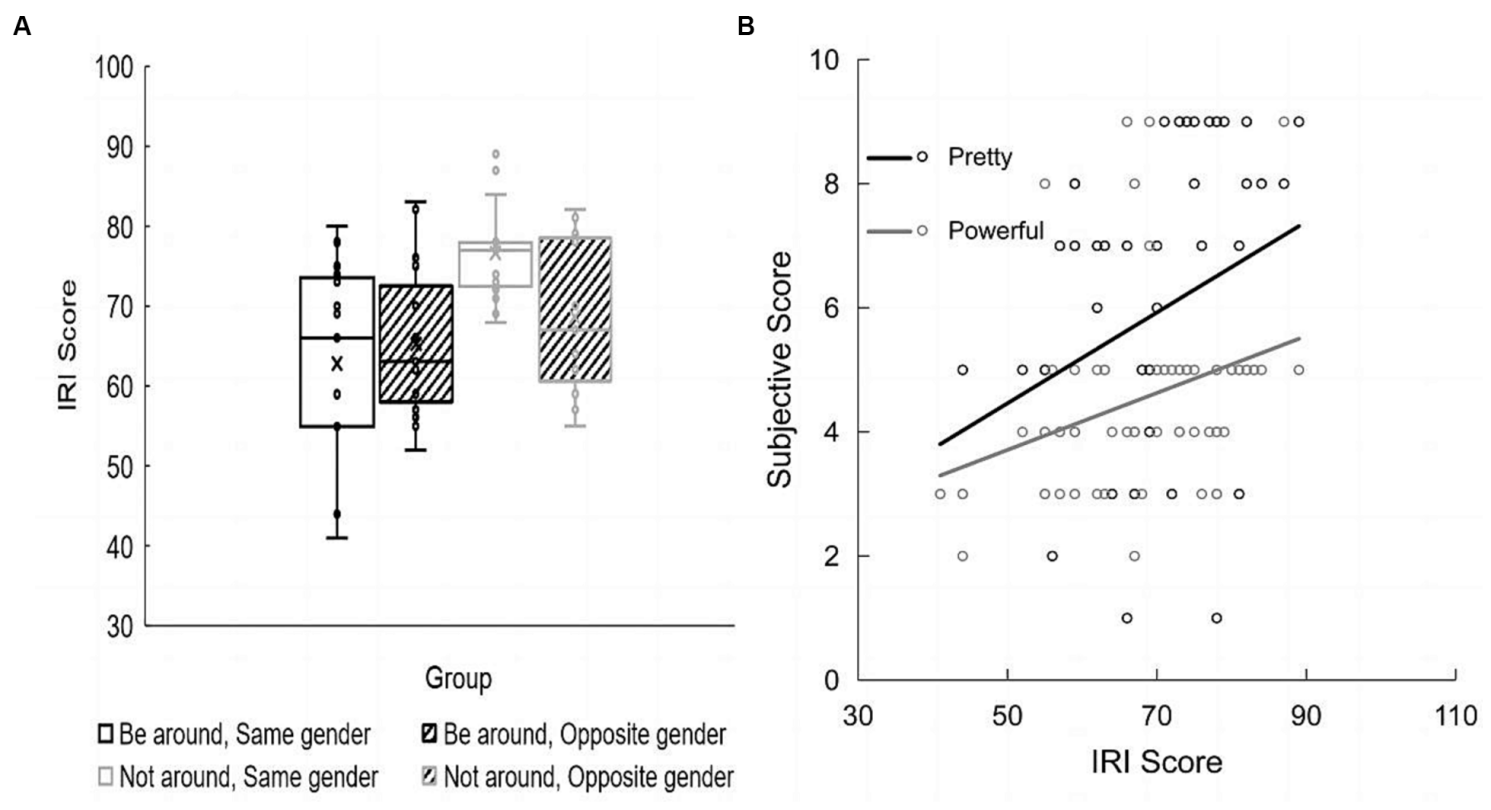
**(A)** Subjective measurement results. The 25th and 75th quartiles and the “whisker” showing the 5th and 95th percentile. **(B)** Subjective measurement correlation. The correlation between IRI score and face value and ability.

Univariate analysis of variance was conducted with partner presence and opponent gender as independent variables and the total score on the IRI scale as the dependent variable. The results showed that when the partner was absent, the IRI total score (*M* = 72.57; *SD* = 8.89) was significantly higher than that when the partner was nearby (*M* = 64.00; *SD* = 11.36) [*F*(1, 56) = 11.20, *p* = 0.001, $\eta_{p}^{2}$ = 0.167]. The main effect of opponent gender was not significant [*F*(1, 56) = 1.23, *p* = 0.273]. The interaction between partner presence and opponent gender was significant [*F*(1, 56) = 4.18, *p* = 0.046, $\eta_{p}^{2}$ = 0.07]. Further analysis showed that the IRI total score (*M* = 76.60; *SD* = 2.56) in same gender dyads was significantly higher than that in opposite gender dyads (*M* = 68.53; *SD* = 2.56) when the partner was absent, *p* = 0.030. When competing with an opponent of the same gender, the IRI total score (*M* = 76.60; *SD* = 2.56) was significantly higher when the partner was absent (*M* = 62.80, *SD* = 2.56, *p* < 0.001) (Figure 5A).

The results of Spearman correlation analysis showed that the perceived ability (*r* = 0.433, *p* = 0.001) and perceived appearance (*r* = 0.348, *p* = 0.006) of opponents were significantly positively correlated with the IRI total score; that is, the subjective impression of the opponents was positively correlated with the degree of empathy in the task (Figure 5B).

## 4 Discussion

This study used EEG hyperscanning technology to analyze the behavior of dyads participating in competitive interactions and the dynamics of brain oscillations and to explore the impact of partner presence on participant competition. Our findings support the research hypothesis and provide evidence for the unique neurodynamic perspective that the presence of intimate partners in a companion state weakens interbrain synchronization during competitive social interactions among females. This enhances our understanding of the complex dynamics of social interactions.

The results showed that when their partner was absent, female participants had higher empathy scores, i.e., higher levels of empathy and shorter reaction times to complete competitive tasks, compared to in the presence of their partner. Previous studies have shown that under competitive conditions, participants reduce inhibitory control and improve their motor responses to defeat opponents, decreasing reaction time (Barraza et al., 2020). This is consistent with the results of the present study. In addition, in this study, in the absence of their partner (when partners are not watching), participants may have been able to focus on competitive tasks without distraction while also paying more attention to their competitors and developing a certain level of empathy (Cazzato et al., 2015; Mehmood et al., 2021). Building upon previous research findings, in interpersonal interactions, empathy-induced positive affect toward competitors may enhance the level of interbrain synchronization and more consistent motor synchronization (Gvirts et al., 2023). This could potentially explain why females exhibit shorter reactions when competing with other females in the absence of their partners.

### 4.1 Late cognitive control affects interbrain synchronization in the Theta frequency band

The present study found that in the late stage of stimulus presentation, in opposite gender dyads with the partner absent, the interbrain synchronization in the Theta frequency band between female participants and their competitors in the inferior parietal lobule (IPL) was significantly greater than that when the partner was present. When the partner was absent and females competed with other females, the interbrain synchronization in the Theta frequency band in the inferior frontal gyrus (IFG) was significantly greater than that when females competed with an opponent of the opposite gender. This finding aligns with the research by Shamay-Tsoory et al. (2019) on the core neural mechanisms of social congruence, which suggests that higher interbrain synchronization in the OE system occurs when there is consistency in movement, emotion, or cognition.

In previous studies, the Theta frequency band has been considered a marker of cognitive load and is closely related to advanced cognitive functions such as cognitive control, memory encoding, and recall (Klimesch, 1999; Jensen and Tesche, 2002). Brain activity in the Theta frequency band reflects an increased demand for specific cognitive functions such as situational memory recall and cognitive control (Babiloni et al., 2009; Sauseng et al., 2010). The IPL is closely related to cognitive functions, such as perspective-taking (Iacoboni and Dapretto, 2006) and self–other differentiation (Jackson and Decety, 2004; Abu-Akel and Shamay-Tsoory, 2011). Compared to cooperation, competition requires additional mental resources, so IPL synchronization is more pronounced in competitive contexts (Liu et al., 2017). In the present study, when their partner was nearby, female participants may have wanted to maintain a good image in front of their partner while completing competitive tasks, thereby suppressing intense and image-damaging competitive behavior and failing to make in-depth evaluations and adjustments to their competitive outcomes in a timely manner in the late stages of competition (Cazzato et al., 2015; Mehmood et al., 2021). When their partner was absent, female participants may have immersed themselves in competitive interactions to the same extent as their competitors, actively recalling competition and related experiences in the late stages of competition and trying to control the competitive situation (Arnott and Elwood, 2008). Furthermore, when partners are not present, females engage in competition with opponents with equal and focused involvement, leading to significantly higher levels of interbrain synchronization near the IFG region, which is closely associated with motor synchronization (Gamliel et al., 2021).

From the perspective of females themselves, compared to single participants, females in a stable relationship exhibit decreased attention to opposite gender interaction partners and increased attention to same gender interaction partners (Karremans and Verwijmeren, 2008). Previous studies have shown that compared to males, females tend to exhibit higher levels of empathy and respect, indicating that their level of empathy may be higher (González Moreno and Molero Jurado, 2022). Furthermore, in another study using hyperscanning, the findings revealed that stronger mutual attention during social interactions leads to enhanced interbrain synchronization among participants, thereby facilitating their ability to engage in specific interactions and better achieve goals (Gvirts and Perlmutter, 2020). This is also confirmed by the results in the subjective evaluation section. Some studies have shown that the Theta band is related to working memory, which transmits stimulus-specific information to the visual cortex during focused attention (Ekstrom et al., 2005). Consistent with these findings, in the present study, the interbrain synchronization of the Theta frequency band was higher in same gender dyads, which may be due to females’s increased attention, greater focus, and increased empathy toward same gender competitors compared to strangers of the opposite gender. This enables female participants to encode information in working memory, adjust and plan their behavior in a timely manner, and win the competition in the late stage after performing competitive key actions.

### 4.2 Interbrain synchronization of the Alpha band as a marker of alertness

Based on previous studies, the Alpha frequency band is closely related to the allocation of attention in selective tasks and the overall alertness of the brain. When alertness decreases, the Alpha frequency band activity increases (Dikker et al., 2020). Song et al. (2022) found that curling athletes exhibit greater brain activity in the Alpha frequency range during competitive tasks than inexperienced participants. This result indicates that the brain activity in the Alpha frequency band is related to factors such as the participant’s competitive experience, alertness toward task stimuli, and visual processing of stimuli.

In this study, in the early stage of competition, when female participants competed with same gender opponents in the partner-absent condition, the Alpha frequency band in the IPL exhibited the highest interbrain synchronization. This may be because, at this stage, cognitive empathy has not yet been deeply processed, and the Alpha frequency band reflects only the results of participant visual processing when viewing the same stimulus (Clements et al., 2023). In same gender dyads, both females can guess each other’s intentions given their shared gender and predict their competitors’ subsequent actions, thus reducing the alertness of participant females and their opponents (Ravreby et al., 2022). Based on the above, the presence of a partner may affect the alertness of female participants when participating in the competition. When in the presence of their romantic partners, females may be more inclined to share moments of relaxation and comfort with their partners, rather than being in a state of competition and vigilance (Bouchard and Saint-Aubin, 2014). On the other hand, when their partners are not around, females are able to quickly enter and adapt to competitive situations without the influence of intimate relationships, which may also contribute to the increased interbrain synchronization.

The results of this study on the correlation between interbrain synchronization and behavioral performance (DRT) showed a significant negative correlation between subjects’ DRT and interbrain synchronization (PLV), indicating that smaller differences in reaction times between subjects were associated with stronger interbrain synchronization between subjects. This trend occurred in the late stage of competition in the Theta band and in the early stage of competition in the Alpha band. This result also confirms the potential impact of focused attention (Ekstrom et al., 2005). When both participants focused on the competitive task, when the target appears, both participants immediately pressed the button in response. Compared to distracted participants who are not focused on the job, participants with higher focus had more similar reaction times and higher levels of interbrain synchronization (Boxhoorn et al., 2022; Bowen et al., 2023). This also indicates that when lovers are not present and participants compete with individuals of the same gender, the synchronization between their brains is stronger, resulting in a higher level of focus on the competitive task and a greater willingness to compete. However, when lovers are present or when competing against opposite-gender opponents, females’ s level of focus is not as high as in the previous scenario.

In addition, based on the subjective results, the IRI total score of participants were higher when their partners were absent, and females were more likely to empathize with same gender opponents. Therefore, in same gender dyads, females may better understand each other’s intentions than when competing with unfamiliar opposite gender opponents, which has been confirmed in previous studies (Pang et al., 2023). participants pay more attention to and empathize with same gender competitors, exhibiting higher interbrain synchronization when competing with same gender participants (Kang et al., 2022). Moreover, from the subjective results, there was a significant positive correlation between the personal impression of competitors and empathy of participants toward competitors. When faced with attractive and capable opposite gender opponents, participants may exhibit more compassion toward their competitors and be more willing to invest more energy in the competition (Balconi et al., 2022).

However, there are also some limitations in this study. First, due to venue limitations, the participants in a relationship recruited in this study were mainly from university campuses, and the participants were also students. Therefore, the research results only apply to social interactions and competitions on university campuses. These results may not be generalizable to working couples, married couples, and elderly participants, etc. Future research is needed with a larger age range of subjects to explore the impact of intimate relationships on competition in different age groups and scenarios. Second, this study did not control for the potential impact of female physiological cycles on competition in participants in romantic relationships. Previous studies have shown that females’s responses to social stress may be influenced by the menstrual cycle (Kirschbaum et al., 1999; Duchesne and Pruessner, 2013; Albert et al., 2015; Banis and Lorist, 2017). In competitive interactions, there is inevitably competitive pressure, and female physiological cycles may exert a particular impact on competitive interactions. Future research should investigate the comprehensive effect of intimate relationships and menstrual cycle stages on females’s social interactions and competitive interactions. Third, this study only evaluated the length of time that intimate partners had been in love and did not measure their level of intimacy or relationship patterns (Rice et al., 2022). Therefore, the impact of their partner’s presence on the interbrain synchronization between females and their opponents may be inconsistent. Future research on intimate relationships should be conducted through questionnaires or interviews to achieve a deeper understanding of the emotional state of participants and control the impact of these variables on the research results.

## 5 Conclusion

In summary, based on the theory of sexual selection, this study examined the neurodynamics of competitive social interactions among participants who were in a relationship through EEG hyperscanning technology. It has been found that the companionship state of intimate relationships weakens the brain synchronization of females during competition. The presence of a romantic partner during competition affects the initial alertness and deep-level cognitive control of female individuals. When accompanied by their partners, females tend to share moments of relaxation and comfort with their partners, whereas in the absence of their partners, females become more fully engaged in the competitive task. In addition, when their partner was absent, females exhibited higher interbrain synchronization with opponents and higher empathy for unfamiliar females compared to unfamiliar males. The application of hyperscanning technology in competitive tasks can provide a more realistic understanding of real-time interpersonal interactions and brain activity in real life scenarios, promote understanding of social interactions related to intimate relationships, and provide cutting-edge insights into the evolution of intimate relationships.

## Data availability statement

The raw data supporting the conclusions of this article will be made available by the authors, without undue reservation.

## Ethics statement

The studies involving humans were approved by Ethics Committee of North China University of Science and Technology. The studies were conducted in accordance with the local legislation and institutional requirements. The participants provided their written informed consent to participate in this study.

## Author contributions

SJ: Conceptualization, Data curation, Formal analysis, Investigation, Methodology, Software, Validation, Visualization, Writing – original draft, Writing – review & editing. YM: Conceptualization, Investigation, Writing – review & editing. YG: Conceptualization, Investigation, Writing – review & editing. LA: Conceptualization, Investigation, Writing – review & editing. LY: Conceptualization, Investigation, Writing – review & editing. HW: Conceptualization, Investigation, Writing – review & editing. YL: Conceptualization, Funding acquisition, Investigation, Resources, Supervision, Writing – review & editing.
